# 78. Impact of Methicillin-Resistant *Staphylococcus aureus* (MRSA) Nares Screen on Vancomycin Utilization for Respiratory Tract Infections

**DOI:** 10.1093/ofid/ofab466.280

**Published:** 2021-12-04

**Authors:** Ashita Debnath, Esther King, Dimple Patel, Pamela Giordano

**Affiliations:** 1 Overlook Medical Center, Summit, New Jersey; 2 Morristown Medical Center, Morristown, NJ

## Abstract

**Background:**

*S. aureus*, including MRSA, is a common colonizer of the nares. Recent data have shown that a negative MRSA nares screen by PCR has a negative predictive value of 98%. This implies that the absence of colonization can significantly reduce empiric vancomycin utilization. This study aimed to determine the utilization of MRSA nares screening on patients receiving vancomycin for respiratory tract infections (RTI) following the addition of the screen to the institutional RTI management guidelines.

**Methods:**

This was a retrospective chart review of adult inpatients presenting to two community-teaching hospitals who were prescribed vancomycin for the treatment of RTIs. Patients were divided into pre-guideline (Jan-Feb 2019), post-guideline 1 (Jan-Feb 2020), and post-guideline 2 (Jan-Feb 2021) groups. The primary endpoint was the difference in percent of vancomycin orders discontinued within 24 hours of a negative screen. Secondary endpoints included the percent of screens ordered, re-initiation of vancomycin within seven days for RTI, and total vancomycin days of therapy (DOT) per 1000 patient days (PD).

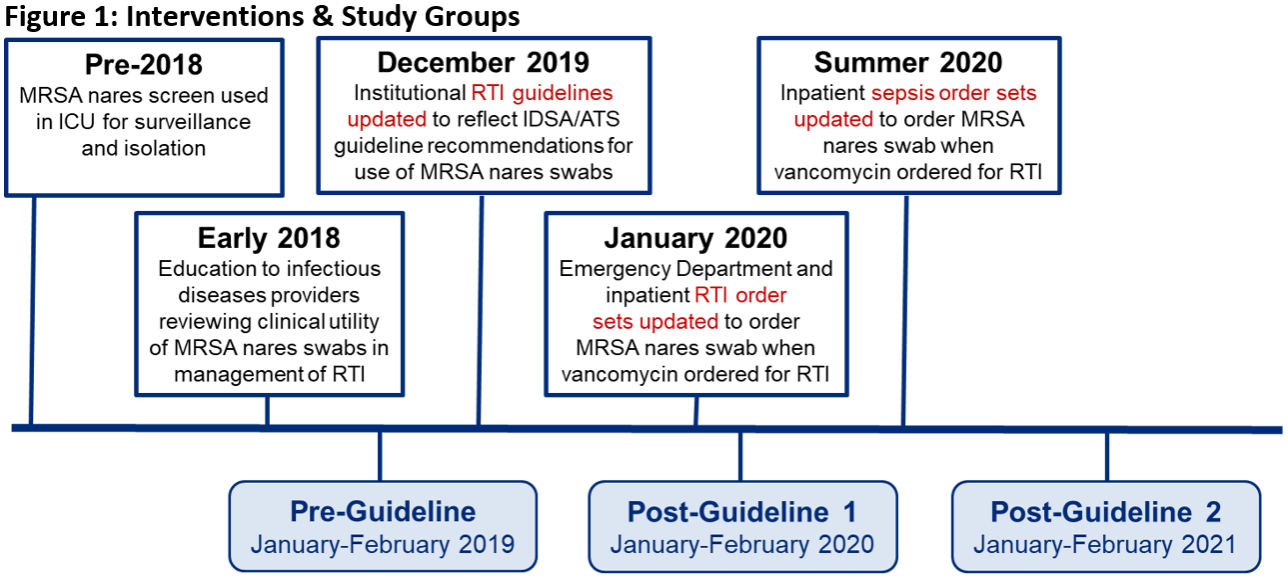

**Results:**

Of 493 vancomycin orders screened, 100 orders in each arm were analyzed. There was an absolute increase of 20.6% in vancomycin orders discontinued within 24 hours of a negative screen between the pre-guideline and post-guideline 2 groups (59.1% vs. 79.7%, *p* = 0.0177). When compared to the pre-guideline group, utilization of the screen increased by 15% in the post-guideline 1 group (48% vs. 63%, *p* = 0.0328) and 26% in the post-guideline 2 group (48% vs. 74%, *p* = 0.000164). There was no difference in re-initiation of vancomycin. A statistically significant reduction in total vancomycin DOT/1000PD from the pre-guideline to the post-guideline 1 and 2 groups (66 to 63 to 60, respectively) was also observed.

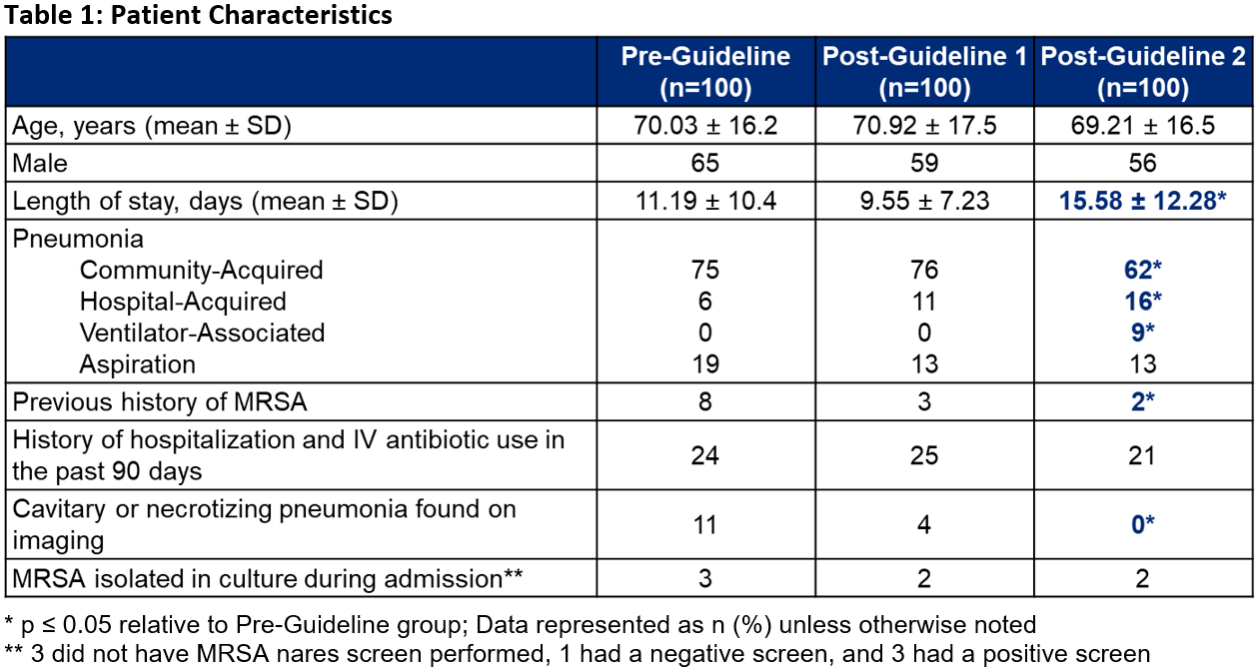

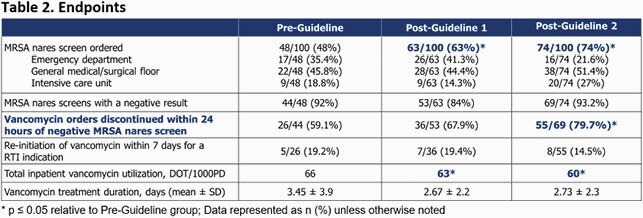

**Conclusion:**

The addition of the MRSA nares screen to the institutional RTI guidelines increased utilization of the test and demonstrated a reduction in vancomycin utilization. With an increase in education, prospective audit and feedback, and prescriber comfort with the use of the MRSA nares screen in the post-guideline 2 group, there was significant improvement in MRSA nares screen utilization, vancomycin discontinuation after a negative screen, and vancomycin utilization.

**Disclosures:**

**All Authors**: No reported disclosures

